# Molecular Epidemiology of the HIV Epidemic in Three German Metropolitan Regions – Cologne/Bonn, Munich and Hannover, 1999–2016

**DOI:** 10.1038/s41598-018-25004-8

**Published:** 2018-05-01

**Authors:** Melanie Stecher, Antoine Chaillon, Josef Eberle, Georg M. N. Behrens, Anna-Maria Eis-Hübinger, Clara Lehmann, Alexandra Jablonka, Johannes Bogner, Gerd Fätkenheuer, Christoph D. Spinner, Jan-Christian Wasmuth, Rolf Kaiser, Sanjay R. Mehta, Joerg Janne Vehreschild, Martin Hoenigl

**Affiliations:** 1 Department I of Internal Medicine, University Hospital of Cologne, Cologne, Germany; 2 German Centre for Infection Research (DZIF), partner site Bonn - Cologne, Cologne, Germany; 3 Division of Infectious Diseases, University of California San Diego, San Diego, CA USA; 4 Max von Pettenkofer Institute & Gene Center, Virology, National Reference Center for Retroviruses, Faculty of Medicine, LMU München, Munich, Germany; 5 German Centre for Infection Research (DZIF), partner Munich, Munich, Germany; 6 Department for Clinical Immunology and Rheumatology, Hannover Medical School, Hannover, Germany; 7 German Centre for Infection Research (DZIF), partner Hannover, Hannover, Germany; 8 Institute of Virology, University of Bonn Medical Centre, Bonn, Germany; 9 Centre for Molecular Medicine Cologne, University of Cologne, Cologne, Germany; 10 Department for Infectious Diseases, Medical Clinic IV, Department of the Ludwig-Maximilians-University Munich, Munich, Germany; 11 Department of Medicine II, Technical University of Munich, Munich, Germany; 12 Department for Internal Medicine I, University Hospital of Bonn, Bonn, Germany; 13 Institute of Virology, University Hospital of Cologne, Cologne, Germany; 14 Division of Pulmonology and Section of Infectious Diseases, Medical University of Graz, Graz, Austria

## Abstract

Using HIV sequence data to characterize clusters of HIV transmission may provide insight into the epidemic. Phylogenetic and network analyses were performed to infer putative relationships between HIV-1 partial *pol* sequences from 2,774 individuals receiving care in three German regions between 1999–2016. The regions have in common that they host some of the largest annual festivals in Europe (Carnival and Oktoberfest). Putative links with sequences (n = 150,396) from the Los Alamos HIV Sequence database were evaluated. A total of 595/2,774 (21.4%) sequences linked with at least one other sequence, forming 184 transmission clusters. Clustering individuals were significantly more likely to be younger, male, and report sex with men as their main risk factor (p < 0.001 each). Most clusters (77.2%) consisted exclusively of men; 41 (28.9%) of these included men reporting sex with women. Thirty-two clusters (17.4%) contained sequences from more than one region; clustering men were significantly more likely to be in a position bridging regional HIV epidemics than clustering women (p = 0.027). We found 236 clusters linking 547 sequences from our sample with sequences from the Los Alamos database (n = 1407; 31% from other German centres). These results highlight the pitfalls of focusing HIV prevention efforts on specific risk groups or specific locales.

## Introduction

Despite ongoing comprehensive prevention efforts, HIV remains one of the most important communicable diseases and a major public health threat in Europe^1^. In Germany, the annual rate of new infections has increased since 2011, with an estimated 3,674 new infections in 2015^2^. The incidence of HIV remains highest in large cities, with Munich and Cologne among the cities with highest incidence rates in Germany, with a prevalence rate of 16.3 and 11.7 per 100,000 respectively^2,3^. The overall number of HIV infected patients is estimated at 84,700 (78,300–91,100) in Germany^4^. Similar to the epidemic across Europe, men-who-have-sex-with-men (MSM) bear the major burden of new infections^1,2,5,6^. Increasing HIV incidence in MSM can be attributable to an increase of risky sex in particular in young MSM, often associated with a simultaneous intake of recreational drugs such as methamphetamine, mephedrone, poppers, or cocaine^7^. Known as “chemsex” or “party and play”, this practice is associated with condomless anal sex, multiple sex partners, and the acquisition of HIV and other sexually transmitted infections (STI) within the home communities^2,8–13^, and during recreational travel^14,15^. Both, rates of recreational substance use and risky sex are particularly high among visitors of large parties and festivals^16,17^, all of which may contribute to bridging regional HIV epidemics within Germany and across Europe^18^.

Since the onset of universal drug resistance testing in Europe^19^, viral sequence availability from HIV infected individuals has dramatically increased. These viral sequences can be used to infer transmission patterns among risk groups and geographical regions and evaluate the dynamics of these regional epidemics^20,21^. These analyses can reveal a more informative picture of the epidemic than standard measures of incidence and prevalence and can guide implementation of prevention measures on a local, national and international level^21–23^.

Here we used molecular epidemiologic analyses to obtain insight into drivers and links between the regional epidemics of Cologne/Bonn, Hannover, and Munich; cities and regions which have in common that they host some of the largest annual festivals in Europe. These include: Carnival and the Christopher Street Day (CSD) in Cologne attracting 1 to 1.5 million visitors each, Oktoberfest in Munich attracting approximately 6 million visitors, and Schuetzenfest and Oktoberfest in Hannover (second largest Oktoberfest in Europe), which attract about 1 million visitors each^24,25^. In a collaborative effort between the institutes of the Translational Platform (TP-HIV) located in Germany and the University of California, San Diego, we evaluated HIV sequence, clinical and socio-demographic data to characterise the transmission network within these three German metropolitan regions and to investigate putative links to publicly available HIV-1 sequences worldwide.

## Results

**Figure 1:**
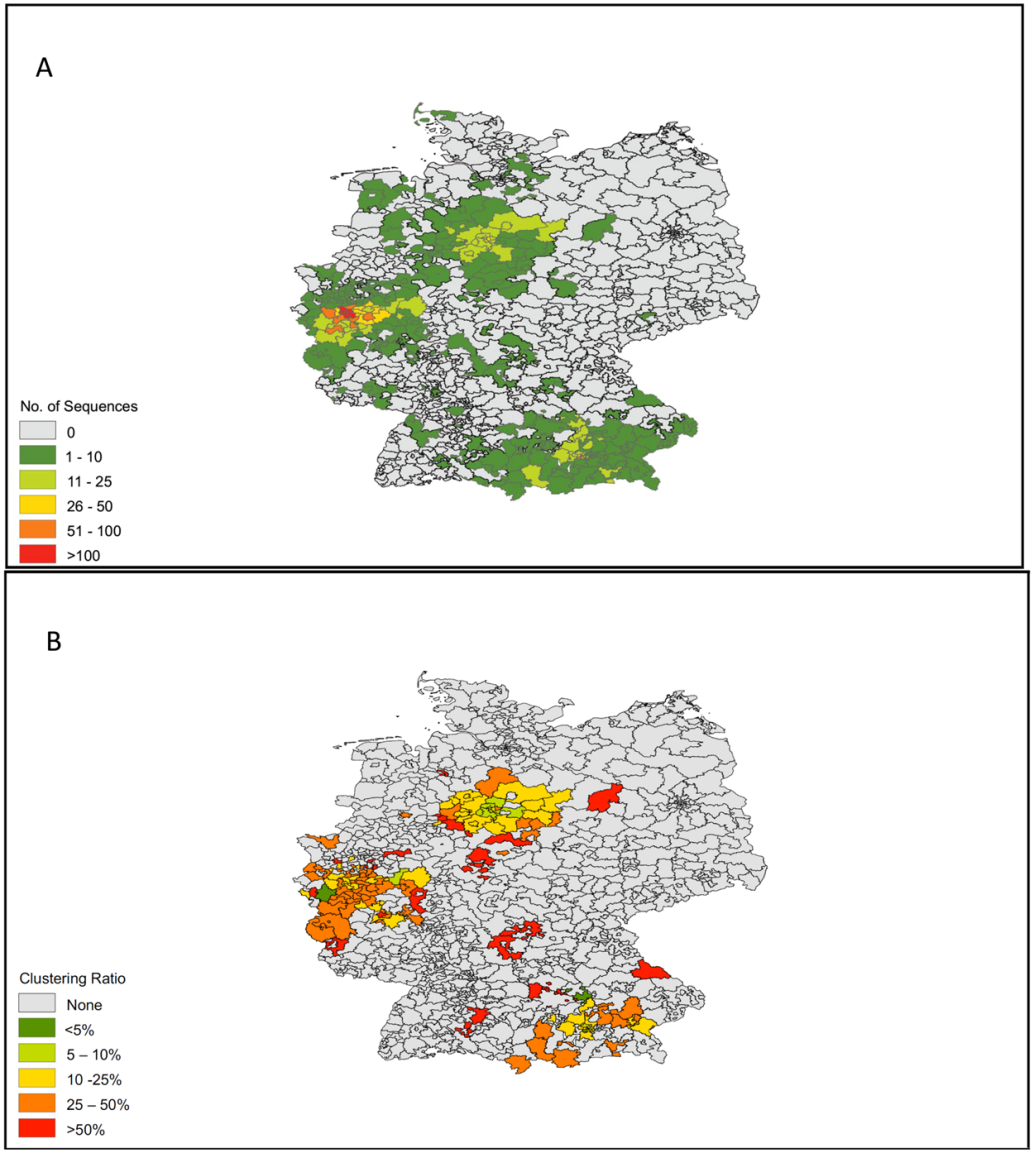
Maps of (**A**) Sampled Population, (**B**) Ratios of Sequences Clustering in each Area. Maps are based on first 3 numbers of zip-codes of residency of reported by participants.

A total of 2,774 participants were included in this analysis. A map demonstrating the geographic distribution of the sampled population is displayed in Fig. 1A. Demographic characteristics, risk factors and HIV subtypes of the sampled population are displayed in Table 1. The sample population was predominantly male (n = 2,222, 80%) with a median age 40 years (IQR 32.5-48). MSM was the most frequently reported primary risk factor (n = 1,448, 52%), followed by heterosexual sex (HTS; n = 622, 22%), origin from a country with >1% HIV prevalence (i.e. endemic region; n = 292, 11%) and injection drug use (n = 137, 5%).

**Table 1:** Population Characteristics.

	Study Sample 1999–2016	Non-clustering	Clustering within the transmission network	Odds Ratio (95% confidence interval)	*P value if* <*0*.*05*
N (%)	2,774 (100%)	2,179 (78.6%)	595 (21.4%)	—	—
***Age (years)***					
Median (IQR)	40 (33–48)	40 (34–49)	36 (30–45)	—	<*0*.*001*
***Sex***					
Male	80.1% (n = 2,222)	77.1% (n = 1,681)	90.9% (n = 541)	1	—
Female	19.6% (n = 545)	22.5% (n = 492)	8.9% (n = 53)	0.33 (0.25–0.45)	<*0*.*001*
Other/NA	0.3% (n = 7)	0.2% (n = 6)	0.2% (n = 1)	0.51 (0.06–4.31)	—
***Region of Origin***					
Germany	69.5% (n = 1,928)	66% (n = 1,439)	82.2% (n = 489)	1	—
Eastern Europe	5.6% (n = 154)	6.2% (n = 136)	3% (n = 18)	0.39 (0.24–0.64)	<*0*.*001*
Western Europe	3% (n = 82)	3.1% (n = 68)	2.4% (n = 14)	0.61 (0.34–1.09)	—
Africa	13.2% (n = 366)	16.1% (n = 351)	2.5% (n = 15)	0.13 (0.08–0.21)	<*0*.*001*
South-East Asia	2.2% (n = 61)	2.2% (n = 47)	2.4% (n = 14)	0.87 (0.48–1.6)	—
Middle-East	2.8% (n = 79)	2.5% (n = 54)	4.2% (n = 25)	1.36 (0.84–2.21)	—
North America	0.3% (n = 7)	0.3% (n = 7)	0%	0.20 (0.01–3.44)	<*0*.*001*
Central-South America	1.4% (n = 39)	1.4% (n = 30)	1.5% (n = 9)	0.88 (0.41–1.87)	—
NA	2.1% (n = 58)	2.2% (n = 47)	1.8% (n = 11)	0.69 (0.35–1.34)	—
***Risk Factor***					
MSM	52.1% (n = 1,448)	48.3% (n = 1,053)	66.3% (n = 395)	1	—
HTS	22.4% (n = 622)	23.3% (n = 509)	18.9% (n = 113)	0.59 (0.47–0.90)	<*0*.*001*
IDU	4.9% (n = 137)	5% (n = 111)	4.3% (n = 26)	0.62 (0.40–0.97)	<*0*.*001*
Endemic (i.e. Origin from Country with HIV prevalence >1%)	10.5% (n = 292)	13% (n = 285)	1.1% (n = 7)	0.06 (0.03–0.14)	<*0*.*001*
Others/unknown	9.8% (n = 275)	10.1% (n = 221)	9.1% (n = 54)	0.65 (0.47–0.90)	*0*.*008*
***HIV Subtype***					
B	73.6% (n = 2,042)	69.2% (n = 1,510)	89.4% (n = 532)	1	—
Non-B	26.3% (n = 732)	30.7% (n = 669)	10.5% (n = 63)	0.27 (0.20–0.90)	<*0*.*001*
***Sampling Date***					
Median (IQR)	2010 (2007–2013)	2010 (2006–2013)	2011 (2009–2014)		<*0*.*001*
**Cohort**					
Bonn	9.3% (n = 259)	7.1% (n = 155)	17.4% (n = 104)	1	—
Cologne	54.3% (n = 1,507)	52.7% (n = 1,150)	60% (n = 357)	0.46 (0.35–0.61)	<*0*.*001*
Hannover	12% (n = 334)	13% (n = 284)	8.4% (n = 50)	0.26 (0.18–0.39)	<*0*.*001*
Munich LMU	23.1% (n = 641)	25.9% (n = 566)	12.6% (n = 75)	0.20 (0.14–0.28)	<*0*.*001*
Munich TUM	1.1% (n = 33)	1.1% (n = 24)	1.5% (n = 9)	0.67 (0.29–1.53)	—

Baseline Demographic, Risk and Viral Characteristics in Clustering versus non-Clustering participants.

Abbreviations: HTS, heterosexual sex; IDU, injection drug use; MSM, men who have sex with men; NA, not available.

### Cluster analyses

**Figure 2:**
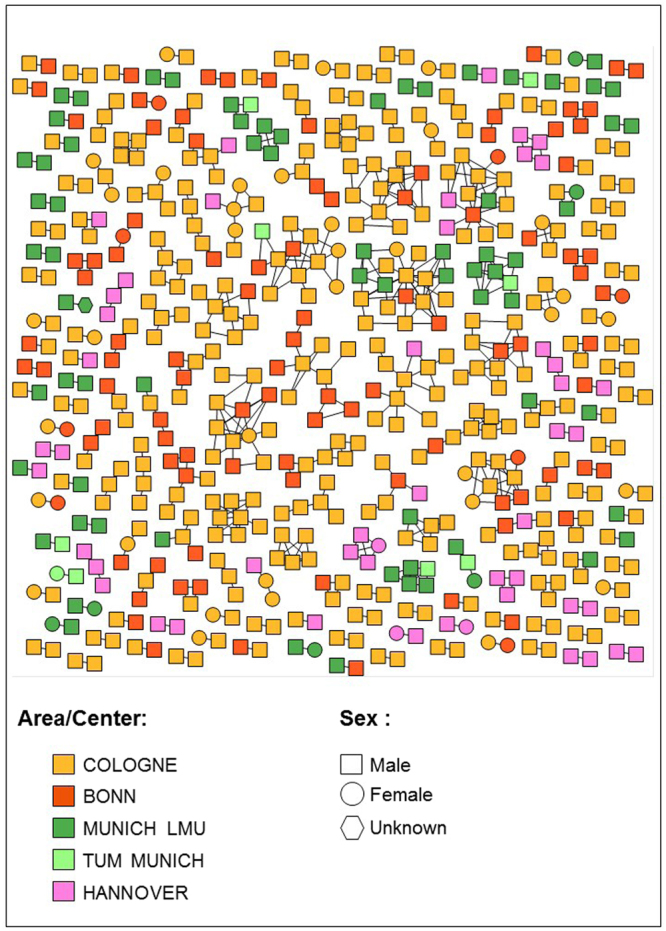
HIV Transmission Network by Center and Region. Individuals (nodes) are shaped as square (men) and circle (women). Nodes are coloured according to the region where they have been identified in yellow (Cologne), red (Bonn), dark green (Munich LMU), light green (TUM Munich) and pink (Hannover). All edges represent a genetic distance ≤1.5% separating nodes.

**Figure 3:**
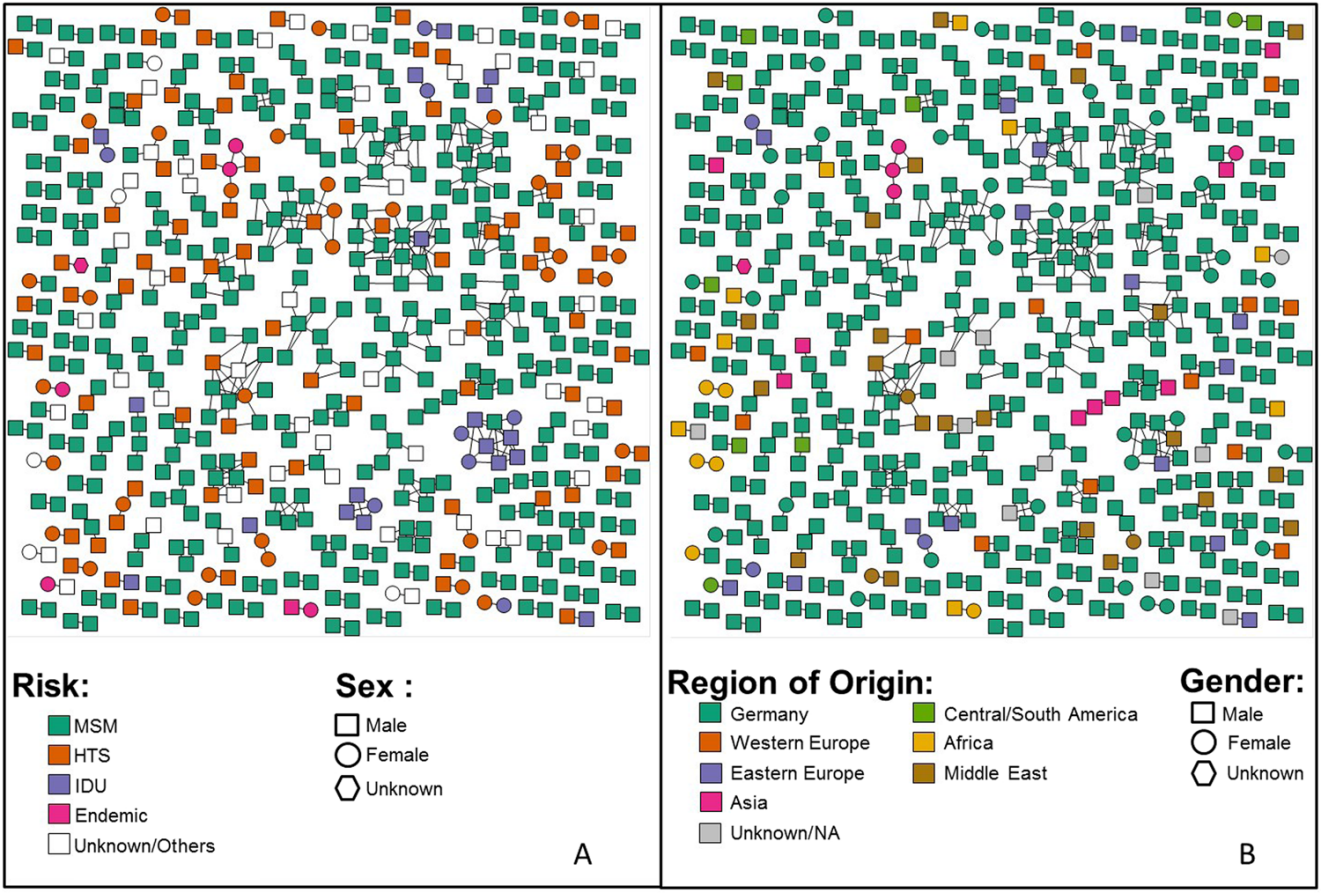
HIV Transmission Network by Risk Factor and Country of Origin. (**A**) Nodes are coloured by their reported risk factor in green (men who have sex with men, MSM), orange (heterosexual [HTS]), purple (injection drug use [IDU]) and pink (endemic), respectively. (**B**) Here, nodes are coloured according to the country/region/continent of origin in green (Germany), orange (Western Europe), purple (Eastern Europe), pink (South-East Asia), yellow (Africa), brown (Middle-East), and red (Central and South America).

A total of 595/2,774 (21.4%) of sequences linked with at least one other sequence, forming 184 transmission clusters, ranging in size from 2 (n = 108 dyads) to 18 sequences. The clustering rates were 12.5% in Munich, 15% in Hannover, and 26.1% in Cologne/Bonn, reflecting the sample densities. A map showing locations of residence for clustering individuals is displayed in Fig. 1B. The majority of transmission clusters [142/184 (77.2%)] were comprised only of men; 41 (28.9%) of these 142 clusters included men who reported sex with women as their primary HIV risk. The HIV transmission network by region is displayed in Fig. 2 and by risk factor and country of origin in Fig. 3A and B.

Mean number of links per clustering node was overall 2.41, and among subgroups 2.46 for males, 1.92 for females, 2.53 for MSM, 2.10 for HTS, 3.19 for IDU, 2.25 for Bonn, 2.62 for Cologne, 1.66 for Hannover, an 2.14 for Munich.

Clustering individuals were significantly more likely to be younger (median age 36 vs 40, p < 0.001), men (90.9% vs 77.1%; χ^2^ = 55.508, p < 0.001), of German origin (82.2% vs 66%; χ^2^ = 57.478, p < 0.001), reporting MSM contact as their main risk factor (66.3% vs 48.3%; χ^2^ = 61.106, p < 0.001), and infected with subtype B (89.4% vs 69.2%; χ^2^ = 97.344, p < 0.001), when compared to non-clustering individuals (Table 1**)**. Individuals below 30 years of age represented 20.8% (n = 124) of clustering individuals and only 10.1% (n = 220) of non-clustering individuals (χ^2^ = 49.664, p < 0.001).

Among individuals reporting heterosexual sex as their main HIV risk factor 113/622 (18.2%) clustered within the network. Men reporting heterosexual sex were significantly more likely to cluster within the network than women (78/355, 22% of men clustered vs 35/266, 13% of women, χ^2^ = 7.936; p = 0.005). Of the 78 clustering men reporting heterosexual sex, 52 (66.7%) were linked to other men but not women, and 34 (43.6%) were linked exclusively with other men reporting MSM contact as their main risk factor.

### Bridging Regional HIV Epidemics

Thirty-two of the 184 transmission clusters (17.4%) contained sequences from more than one region (in addition 42 clusters contained sequences from both Cologne and Bonn, two cities 30 km apart that were defined as one single region for this analysis). Among the 32 transregional clusters, 18 transmission clusters contained sequences from Cologne/Bonn and Munich, 11 from Cologne/Bonn and Hannover, 2 from Munich and Hannover, and one from all three regions. The 32 transregional clusters consisted of 142 individuals, of which 131 (92.3%) were male, 112 (78.9%) reported MSM contact as their primary risk factor and 37 (26.1%) were below 30 years of age.

Next we identified individuals linked to epidemics from other regions, putatively representing individuals that bridged two or more epidemic. Among the 53 women who clustered within the transmission network overall, only four (7.5%) were linked with sequences from other regions (i.e. total 4 edges; all four were linked with each one man from outside the region). In contrast, 107/540 (19.8%) of sequences from clustering men had links with other regions (all but 4 were male to male links); MSM was the primary risk factor in 90/107 (89%) these men. Out of 75 edges linking sequences from different regions (i.e. potentially representing transmissions bridging regional HIV epidemics), 61 (81.3%) linked two MSM, and 8 (10.7%) linked a MSM with a man reporting heterosexual sex as his main risk factor. Edges from clustering men were significantly more likely transregional (11% of edges transregional) than edges from clustering women (3.9% of edges transregional; p = 0.027; Fishers exact test).

### Cluster growth

The 8 large clusters containing more than 10 individuals consisted mainly of MSM, and the majority of 60/108 (55.6%) of individuals in these clusters were men below 40 years of age. The cluster with the fastest recent growth (i.e. 5 newly diagnosed infections over last 2 years), was the only cluster containing sequences from all three sampled regions and consisted of 13 MSM and one heterosexual woman.

### Links between the Regional Epidemics and the Global HIV Epidemic

**Figure 4:**
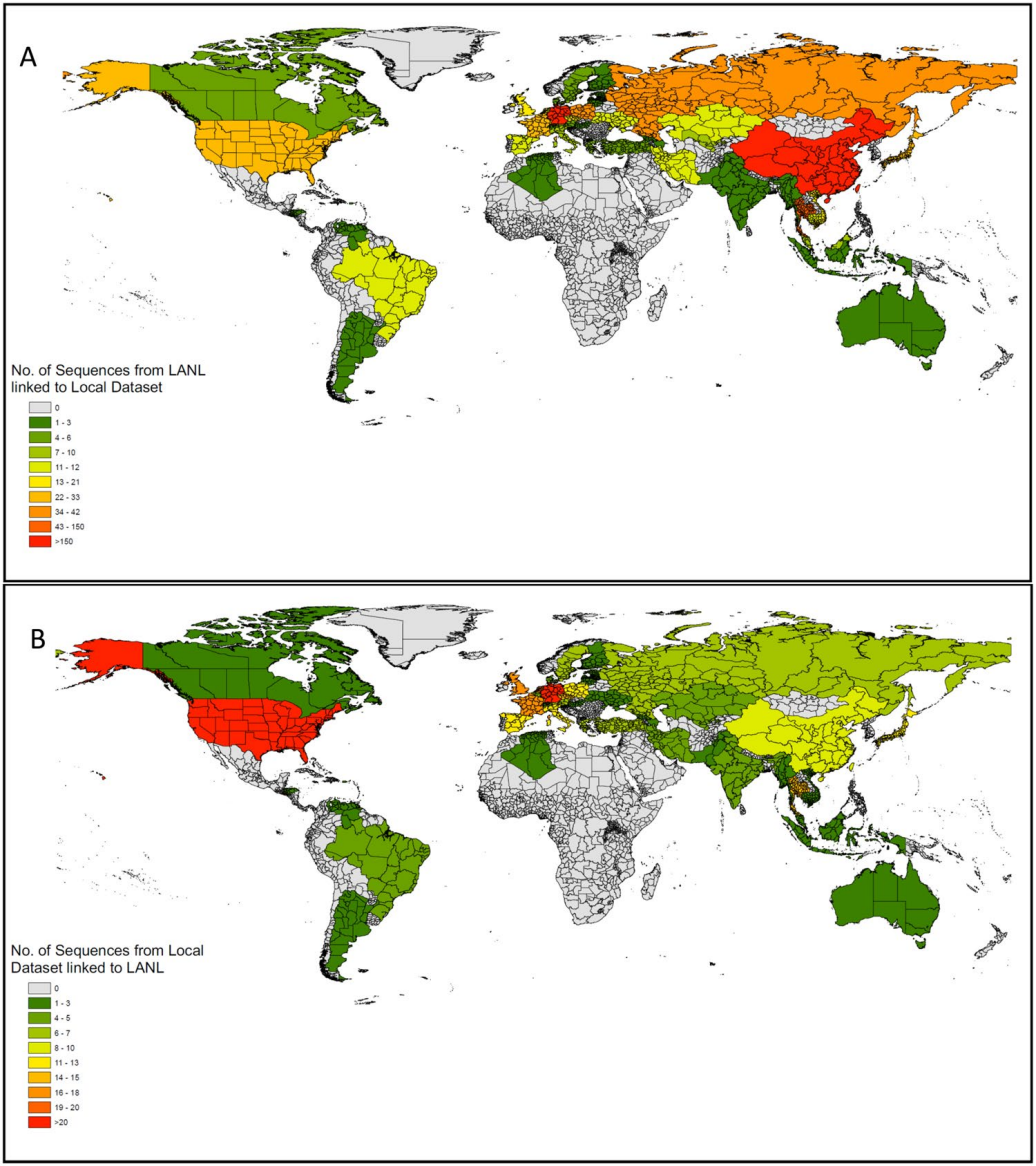
Maps showing linkage with publicly available sequences worldwide. (**A**) Heatmap showing per country number of publicly available sequences linked to this German dataset. (**B**) Heatmap showing per country number of sequences from this German dataset linked to publicly available sequences worldwide.

By combining the 2,774 sequences from Cologne/Bonn, Munich and Hannover with 150,396 publicly available HIV polymerase sequences, we found a total of 236 clusters that included both, our sequences [total n = 547; 432/547 (79%) from Cologne and 91/574 (16.6%) from Munich] and publicly available sequences [total n = 1407; 437 (31.1%) from other German centres, 281 (20%) from other European countries, 356 (25.3%) from China, 175 (12.4%) from South-East Asia and 54 (3.8%) from America]. Figure  4A and B show maps demonstrating the number of publicly available sequences linked to this German dataset, and the number of sequences in this dataset linked to the LANL database.

## Discussion

To our knowledge, this is the largest study to date focusing on the HIV-1 transmission network in Germany, including a total of 2,774 HIV-1 sequences from unique individuals, of which 21.4% linked with at least one other sequence, forming 184 transmission clusters. Two major findings are evident. First, the HIV-1 epidemics in Cologne/Bonn, Munich and Hannover, three German metropolitan regions with particularly high rates of new HIV diagnoses, were not only interlinked, but also linked to epidemics across Germany and around the world. After correcting for representation, men were still more likely to be found in a position bridging regional HIV epidemics than women. Second, discordant links between HIV risk groups were frequent and mostly observed between men reporting heterosexual sex as their primary risk and men reporting MSM contact.

While the majority of clusters were regional clusters, 32 (17.4%) of transmission clusters contained sequences from multiple regions sampled in this study. In addition, four of the 8 large and actively growing clusters, including the cluster with the fastest recent growth, were transregional clusters, containing sequences from two or more metropolitan regions. While one could speculate that transmissions occurring during large festivals within the sampled regions, where young men represent the majority of visitors^24,25^, could have contributed to these viral transmission links between regions across Germany and the world, our results should be interpreted with caution, as data from regions with no large festival activities were not included in our analysis. While the data that was available from participating centers did not allow us to specifically relate new HIV infections to specific festivals, previous studies indicate that both rates of heavy alcohol and substance use and subsequent condomless sex are alarmingly high among visitors of large parties and open air festivals^17,26^, and may result in increased transmission of sexually transmitted infections and also HIV^16^. Statistics from these large German festivals, which attract crowds from across Germany and the world, indicate that young (from 1999 to 2014 between 43% and 60% of visitors of the Munich Oktoberfest were below 30 years of age) men (between 49 and 62% of visitors of the Munich Oktoberfest were male) represent the majority of visitors^24,25^. In this study men below 30 years of age were significantly more likely to cluster within each region and with individuals from other regions, and even after correcting for gender specific clustering rates and number of links, men were significantly more likely to be found in a position bridging regional HIV epidemics than women. While the contribution of large festivals (versus e.g., group sex parties, and one on one contacts during travel) remains speculative, our findings indicate that younger men including MSM, who are more prone towards taking risks, use drugs, and travel^12,14,27^, may be driving the migration of virus from one region to another.

Men below 40 years of age composed also the majority of nodes in large and growing transmission clusters in this study. Growing clusters are an ideal target for prevention interventions, such partner services^28^ for identification of sexual and social partners, partner testing for acute HIV infection^28^, immediate ART in those diagnosed with HIV^29^ and immediate pre-exposure prophylaxis (PrEP) in socially-linked individuals who test negative^30^. All of these may be effective measures to interrupt cluster growth and reduce transmission events. The finding that as many as half of the growing clusters consisted of sequences from more than one metropolitan region, highlights the pitfalls of focusing prevention efforts on specific regions, and emphasizes the need for transregional strategies and collaborations in order to effectively battle the HIV-1 epidemic, and address the increased risk behaviour commonly seen during travel. In addition to multiple links between sequences from Cologne/Bonn, Munich and Hannover, we found also high rates of clustering with sequences from other German centres and European countries, confirming that the viral spread in European countries is highly interconnected^31,32^.

Nearly 80% of transmission clusters identified in this study consisted exclusively of men. MSM bear the major burden of HIV infections in Germany^2^, and MSM had a significantly higher likelihood of clustering within the transmission network in our study, which is similar to other European countries^21,32,33^. More surprising was our finding that two-thirds of the 78 men who clustered within the network and reported heterosexual sex as risk factor only had direct links with sequences from other men, and again two-thirds of those had direct links exclusively with MSM. Importantly, the inferred transmission links in this study are only putative, as additional unsampled individuals could have been intermediary members of the transmission chain linking the two linked individuals^34^. Therefore, we can’t rule out that there might be “missing” women and/or men with multiple risk factors (e.g. heterosexual/MSM [Bisexual], or MSM/IDU) bridging some of our sampled HIV-1 infected MSM and heterosexual males^21^. However, the exceptionally high proportion of self-reported heterosexual men who clustered only with men suggests misrepresentation of risk, potentially related to perceived stigma that prevents males from self-identifying as MSM. These potential nondisclosed MSM^35^ have recently been reported in a study from the United Kingdom to have fewer partners than MSM and to preferentially partner with other potential nondisclosed MSM^35^. That study also suggests that potential nondisclosed MSM have a higher risk for HIV than male heterosexuals and may put female partners at risk by linking the MSM and heterosexual epidemics^35^.

The study is subject to important limitations, many of which refer to the fact that, naturally, transmissions networks are incomplete because they do only include HIV infected persons who get diagnosed within the particular regions that have been sampled, and will never include HIV-infected persons who have not yet been diagnosed. Further the proportion of individuals included in this study versus all newly HIV diagnosed individuals in the respective cities varied between 20% and 90%, explaining differences in clustering rates between the participating centers. Similiar to other studies estimation of sampling density in our study was limited by being based on number of new HIV diagnoses and not based on actual incidence of new HIV infections^36^. Overall, the clustering rates reported here are comparable to those reported in previous studies^35,37^.

Other limitations of the study include a potential sampling and selection bias, meaning that the sequences included in this analysis may not be fully representative of the actual HIV epidemics (e.g. because men and MSM were more likely to be tested for HIV at the participating centres than women). We deem this unlikely, however, as demographic characteristics were relatively constant over participating hospitals and also match well with other data published on the German HIV epidemic. Our dataset is limited to sequences that were available from four University Hospitals located in Cologne, Bonn, Munich and Hannover. A more comprehensive sample from other regions in Germany would have probably shown more transmission links, a higher clustering rate, and thus a better picture of the transmission network throughout the country. Despite this limitation, the population was large and covered geographical regions in the north, west and southern parts of Germany. Also we were not able to draw any conclusions regarding the role of the large annual festivals in HIV transmission and it may be equally likely that other factors, such as e.g. party venues available in those cities all-year around, may have contributed to linkage between this regional epidemics.

In conclusion, our results highlight the pitfalls of focusing prevention efforts on specific risk groups or specific locales, and not taking into consideration viral migration. Misrepresentation of risk may be frequent among men reporting heterosexual sex as their main risk factor. HIV prevention programs should develop effective approaches to target disclosed and non-disclosed sexually adventurous gay and bisexual men who may contribute significantly to bridging different HIV epidemics. Future studies should focus on real time monitoring of cluster growth and the influence of travel and migratory events as well as the party and play culture.

## Methods

The study was designed as a multi-centre project performed by the TP-HIV by the German Centre for Infection Research (DZIF) (NCT02149004), approved by the scientific steering committee of all partner sites, and designed and completed in close cooperation with the University of California, San Diego. The study and protocols were approved by the Ethics Committees of the University Hospitals of Bonn and Cologne, the Hannover Medical School and by the Technical University- and the Ludwig-Maximilian University in Munich, and an informed consent was obtained from all participants. De-identified data (i.e. not attributable to individual patients) was obtained from five partner sites of the DZIF, located in the north-, west- and southern parts of Germany: University Hospital Cologne, University Hospital Bonn (Bonn and Cologne are only 30 km apart forming 1 metropolitan region), Technical University of Munich, Ludwig-Maximilian University Munich, and Hannover Medical School. The methods were carried out in accordance with the approved guidelines.

### Study Population and Sequence Analysis

The study population included 2,774 unique HIV-1 infected individuals enrolled between 1999 and 2016. Analysis was performed using one HIV-1 partial *pol* sequence from each unique individual (HXB2 position 2550 → 3356) obtained at the first HIV clinic visit. All sequences were subtyped using the Subtype Classification using Evolutionary Algorithms (SCUEAL) program^38^.

Viral sequences were included from the University Hospital of Cologne (n = 1,507), University Hospital Bonn (n = 259), Ludwig-Maximilian University Munich (LMU; n = 641), the Interdisciplinary HIV Centre Technical University Munich (TUM; n = 33), and the Hannover medical school (n = 334). A standardized format was used to collect data from each partner site and was merged into a common dataset including socio-demographic, clinical data and viral sequences. Data collected (all from the time of sampling via face-to-face interviews) included age, sex, first three digits of the zip-code of residency, first positive HIV test, HIV subtype, main risk factor for acquiring HIV infection, and the country of origin.

The sampled population was comparable in terms of demographics to the overall population of individuals with new HIV diagnoses in the four cities^38^. The number of newly diagnosed HIV cases in the four cities was collected since 2001 and was 2,177 for Cologne, 266 for Bonn, 2,551 for Munich and 834 for Hannover^38^, indicating that this study (sequences sampled between 1999 and 2016) included about 60–70% of newly HIV diagnosed individuals from Cologne, 85–98% from Bonn, 23–26% from Munich, and 36–40% from Hannover.

### Genetic Network Analysis

We used HIV-TRACE software (HIV TRAnsmission Cluster Engine; www.hivtrace.org) to infer transmission clusters as previously described. All partial HIV *pol* sequences were aligned to the HXB2 reference sequence. Putative transmission links (i.e., edges) were inferred when two sequences (i.e. nodes) had a Tamura-Nei 93^39^ genetic distance of ≤1.5%. The ≤1.5% threshold has been established as a standard in the field, and is based on study findings showing that within mono-infected persons, *pol* sequences typically do not diverge more than 1% during the first 10 years of infection^40,41^. All nucleotide ambiguities were resolved and only sequences with less than 1.5% diversity were retained^42^. Multiple linkages were then combined into putative clusters. Clusters comprised of only two linked individuals were identified as dyads. We repeated the analysis after excluding codons associated with major drug resistance mutations to assess for potential impact of convergent evolution^43^.

### Links between the Regional Epidemics and the Global HIV Epidemic

To better understand the intersection between the HIV Epidemic in the three metropolitan regions Cologne/Bonn, Munich, Hannover and the national and global HIV epidemic, we applied the network-based approach described above to infer all putative links between the 2,774 sequences sampled in this study and all publicly available HIV *pol* sequences in the Los Alamos National Laboratory HIV sequence database (n = 150,396)^44^.

### Statistical analysis

Socio-demographic and medical data were displayed as absolute numbers plus percentages, medians plus interquartile ranges (IQR) or means plus 95% confidence intervals (95%CI), as appropriate. Characteristics between participants clustering and non-clustering in the transmission network were compared using the Fishers exact or Chi-squared test and calculating odds ratios for categorical and the Mann-Whitney-U test for continuous variables. Normally and not normally distributed data was analysed by parametric and non-parametric tests. Statistical analyses were completed by using SPSS statistics software, version 23 (IBM Corp., Armonk, NY, USA).

### Data availability

All sequences were made publicly available. The GenBank accession numbers for the pol sequences included in this analysis are MH071992-MH074765. The datasets used and/or analysed during the current study are available from the corresponding author on reasonable request.

### Ethics approval and consent to participate

The study and protocols were approved by the local Ethics Committees of the University Hospitals of Bonn (reference number 279-14) and Cologne (reference number 13-364), the Hannover Medical School (reference number 2411-2014) and by the Technical University- (reference number 438-14) and the Ludwig-Maximilian University in Munich (reference number 459-14). and individuals gave written informed consent.
